# Treatment approaches for multiple Myeloma vertebral column lesions – Results from an international survey distributed to the AO spine knowledge forum tumor

**DOI:** 10.1016/j.bas.2025.104387

**Published:** 2025-08-06

**Authors:** Vanessa Hubertus, Emily J. von Bronewski, Lucius S. Fekonja, Anton M. Früh, Christian J. Entenmann, Hannah Miller, Charlotte Buhre, Michael G. Fehlings, Ilya Laufer, Peter Vajkoczy, Julia S. Onken

**Affiliations:** a Department of Neurosurgery, Charité – Universitätsmedizin Berlin, Corporate Member of Freie Universität Berlin, Humboldt-Universität zu Berlin, And Berlin Institute of Health, Germany; b BIH – Berlin Institute of Health Charité Clinician Scientist Program, Berlin Institute of Health, Berlin, Germany; c Division of Neurosurgery and Spinal Program, Toronto Western Hospital, Krembil Neuroscience Center, and University of Toronto, Toronto, Canada; d Spine Tumor Program, New York University, New York, United States; e German Consortium for Translational Cancer Research, DKTK, Part of the German Cancer Research Centre, Berlin, Germany

**Keywords:** Multiple Myeloma, Vertebral column lesions, Chronic vertebral pain, Surgical decision-making, International treatment standards

## Abstract

**Introduction:**

Vertebral fractures and epidural compression are common complications in Multiple Myeloma (MM). Although non-surgical management is generally preferred, internationally accepted management guidelines are lacking. This study aimed to assess current international treatment approaches and clinical conditions guiding decision-making in MM vertebral lesions.

**Research question:**

Assessing international treatment standards for MM vertebral column lesions.

**Material and methods:**

A survey was distributed to members of the AO Spine Knowledge Forum Tumor, an expert forum specialized on the treatment of oncologic spine disease. The survey consisted of 25 questions, of which 15 assessed the participant's background, clinical expertise, and experienced treatment standards regarding MM vertebral lesions, followed by ten fictional case examples with seven possible treatment scenarios each.

**Results:**

51 international experts completed the survey, 51 % being of orthopedic, and 44 % of neurosurgical background, while 5 % were radio-oncologists. 84 % of the participants stated they “see vertebral lesions in MM in general as a non-surgical disease”. As strongest indicators to perform surgery, neurological deficits (74 %), and potentially unstable lesions (20 %) were chosen. Clinical and radiological follow-up is performed by 83 %, however only in 46 % at defined intervals. 89 % would choose “less invasive” surgical strategies in MM than in similar lesions related to metastatic spine disease.

**Discussion and conclusion:**

The participating experts agreed towards a more restrained and less invasive management of MM patients, however the applicability of surgical scores, standards for follow-up, and indications as well as surgical strategies for MM vertebral lesions varied widely, illustrating the need for international guidelines standardizing treatment.

## Highlights

• 84 % of 51 international experts stated they “see vertebral lesions in MM in general as a non-surgical disease”.
• As strongest indicators to perform surgery, neurological deficits (74 %), and potential instability (20 %) were chosen.
• The applicability of surgical scores, standards for follow-up, and indications as well as surgical strategies for MM vertebral lesions varied widely, illustrating the need for international guidelines standardizing treatment.

## 1 Introduction

Multiple Myeloma (MM) as a plasma cell malignancy is frequently associated with vertebral column lesions. Next to acute or chronic vertebral pain, epidural compression and neurological deficits are frequent complications during the disease course (Callander et al., 2022; Edwards et al., 2008; Castella et al., 2018; Amelot et al., 2016; Gokaraju et al., 2016; Burks et al., 2021; Panaroni et al., 2017).

Non-surgical management of MM vertebral column lesions is generally preferred due to the fear of surgical complications related to poor bone quality and a higher likelihood of surgical site infections in immunocompromised patients (Callander et al., 2022; Burks et al., 2021; Milavec et al., 2020a; Bilsky and Barzilai, 2021; Shen et al., 2018; Eseonu et al., 2023; Kyriakou et al., 2019; Amelot et al., 2023; Osterhoff et al., 2023). As MM is considered radiosensitive, lesions with or without epidural tumor extension are often and effectively managed with radiotherapy and systemic therapy (Callander et al., 2022; Bilsky and Barzilai, 2021; Osterhoff et al., 2023; Miller et al., 2017; Terpos et al., 2016; Lang et al., 2017; Zijlstra et al., 2023a, 2024; Puvanesarajah et al., 2015; Bingham et al., 2017).

Despite the tendency to manage MM vertebral lesions nonoperatively, a number of patients will receive spine surgery either initially, or during disease progression, either due to otherwise non-manageable pain, (potential) spinal instability, or neurological deficits. Nonetheless, internationally accepted guidelines when and how to operate on MM vertebral column lesions are lacking. Within their latest consensus statement, the International Myeloma Working Group (IMWG) provided an algorithm for treatment decisions in respective patients, naming surgery as an option for lesions with soft tissue involvement and no clinical improvement on radio-/chemotherapy and steroids (Kyriakou et al., 2019). Although several studies could show improved pain scores after surgical management or bracing in those patients, the level of evidence is low, and mostly created by cohort studies and case series (Kyriakou et al., 2019; Cawley et al., 2019; Malhotra et al., 2016; Jordan et al., 2014).

The aim of this study was to assess the current treatment approaches and clinical conditions guiding surgical decision-making in MM vertebral lesions, as they are perceived by an internationally leading group of clinical experts. This was undertaken by distributing a survey to the members of the AO Spine Knowledge Forum Tumor, an international, interdisciplinary expert forum focused on the treatment of oncologic spine disease.

## 2 Material and Methods

### 2.1 Survey creation

A survey was created and distributed to the members of the AO Spine Knowledge Forum Tumor, an international, interdisciplinary expert forum specialized on the treatment of oncologic spine disease. The survey consisted of 25 questions in total, of which 15 were general questions assessing the participant's background, clinical expertise, and their personally experienced treatment standards regarding MM vertebral lesions, followed by ten case examples with the choice between seven standardized treatment scenarios each. In the creation of the ten case studies, medical illustrations modelled by actual patients' MRI and/or CT imaging were created using Adobe Creative Cloud Photoshop, to unify imaging quality, minimize distractions, and create representative illustrations. In all scenarios, seven identical standard treatment options were given to choose from (“no surgery – conservative treatment only”, “no surgery – referral to radiotherapy”, or “surgery - cement augmentation”, “surgery - posterior decompression without instrumentation”, “surgery - posterior decompression with instrumentation”, “surgery - 360° reconstructive spine surgery”, “surgery - separation surgery with adjuvant SRS (stereotactic radiosurgery) or CBRT (conventional beam radiation therapy)”).

### 2.2 Survey distribution, data management and statistical analysis

After its creation, the survey was integrated into a SurveyMonkey questionnaire database and distributed with the support of AO Spine to the 65 members of the AO Spine Knowledge Forum Tumor. The survey remained online for a total of four weeks, with two reminders being sent to the participants before completion. Anonymized data was extracted, and data analysis and visualization were performed using R with R Studio, Version 2023.06 (used packages: ggplot2, dplyr, redxl), and biorender.com. All statistical analyses were of exploratory nature.

### Ethical statement

This study was conducted in accordance with the ethical principles of medical research according to the Declaration of Helsinki and its later amendments. Clinical case examples were purely fictional, and participant's answers completely anonymous. No clinical patient data were collected or distributed.

## 3 Results

### 3.1 General characteristics of the survey participants

**Fig. 1:**
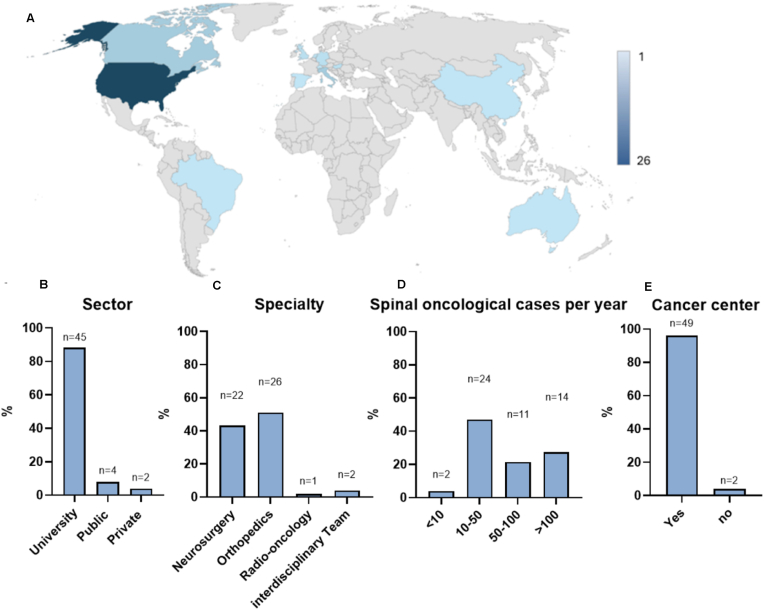
General characteristics of the survey participants, with A) geographical localization, B) associated health-care sector, C) specialty background, D) treated spinal oncology cases per year, E) association to a specialized cancer center. Values are given in total numbers (n) and percentage (%), with n = 51 equaling 100 %.

A total of 51 international experts completed the survey (response rate: 78 %). The experts originated from 14 countries and five continents (Fig. 1A). Regarding medical specialties, 51 % were of orthopedic, 44 % of neurosurgical, and 5 % of radio-oncological background. Regarding their affiliated health-care sectors, 87 % of the participants were university-associated, while 96 % were part of interdisciplinary cancer centers at their affiliated institution, comprising of spine surgeons, radio-oncologists, and medical oncologists. Regarding the number of spinal oncological cases performed at their affiliated institutions, 27 % were part of high-volume centers performing 100 or more cases per year, while 22 % performed between 50 and 100 cases/year, and 51 % less than 50 (Fig. 1B–E).

### 3.2 Perceived treatment standards in Multiple Myeloma vertebral column lesions

**Fig. 2:**
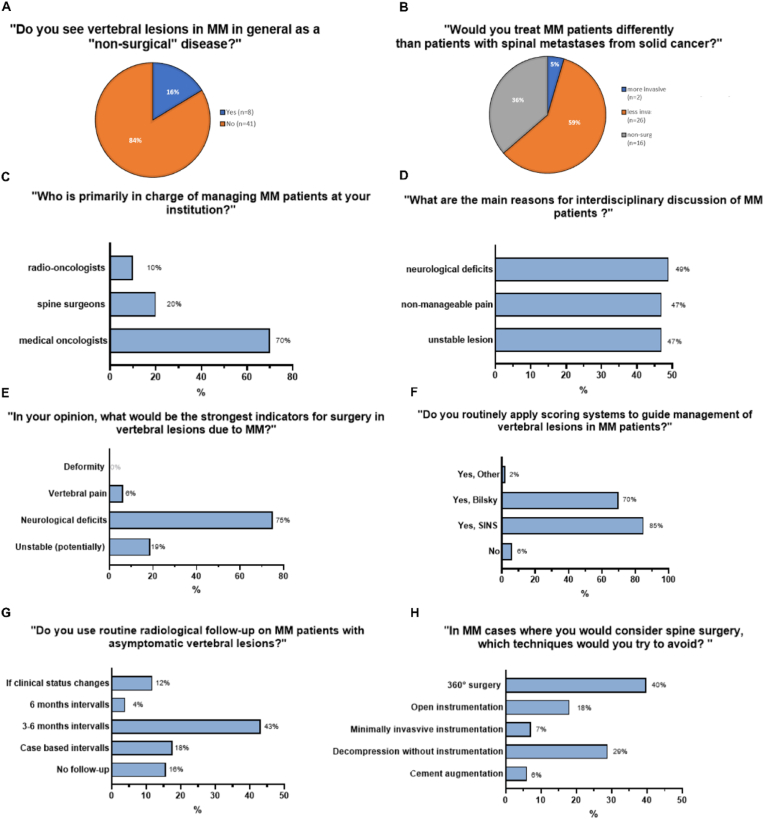
Examples of the participant's answers regarding their perceived treatment standards for Multiple Myeloma vertebral column lesions, with answers to the questions A) “Do you see vertebral lesions in MM in general as a “non-surgical” disease?, B) “Would you treat MM patients differently than patients with spinal metastases from solid cancer?”, C) “Who is primarily in charge of managing MM patients at your institution?” D) “What are the main reasons for interdisciplinary discussion of MM patients?”, E) “In your opinion, what would be the strongest indicators for surgery in vertebral lesions due to MM?”, F) “Do you routinely apply scoring systems to guide management of vertebral lesions in MM patients?”, G) “Do you use routine radiological follow-up on MM patients with asymptomatic vertebral lesions?” and H) “In MM cases where you would consider spine surgery, which techniques would you try to avoid?”. Values are given in percentage (%), with n = 51 equaling 100 %.

**Fig. 3:**
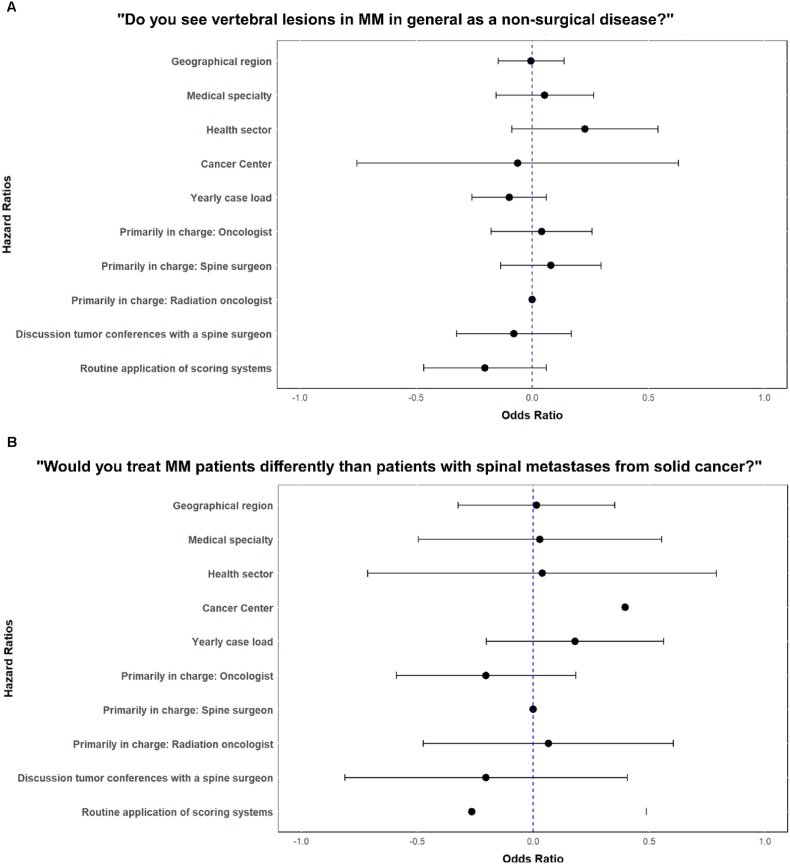
Odd's Ratio of factors associated with the participant's answers. A) to the question if they “see vertebral lesions in Multiple Myeloma in general as a “non-surgical” disease?”, B) to the question if they “would treat Multiple Myeloma patients differently than patients with spinal metastases from solid cancer”. Odd's Ratio is given from −1 to +1, calculated using R with R studio.

When generally asked, 84 % of the participants stated that they “see vertebral lesions in MM in general as a non-surgical disease”. The specialists primarily in charge at their institutions for managing those patients were mostly medical oncologists (70 %) and less commonly spine surgeons (20 %) or radio-oncologists (10 %). Routinely discussed in interdisciplinary tumor boards were MM patients with vertebral lesions in 47 %, while the main reasons resulting in interdisciplinary discussions were given as neurological deficits (49 %), non-manageable pain (47 %), and (potentially) unstable spine lesions according to a radiologist (47 %). As strongest indicators to perform surgery, neurological deficits (74 %) and potentially unstable lesions (20 %) were chosen, while non-manageable pain was named in 6 %. As scores that were routinely applied to guide surgical decision-making, the SINS (Fisher et al., 2010, 2014) (85 %), and Bilsky (Bilsky et al., 2010) (70 %) scores were most frequently named, while single participants additionally named the Enneking, SORG (the Skeletal Oncology Research Group algorithm), and Karnofsky (KPS, Karnofsky Performance Scale) scoring systems (Tokuhashi et al., 2017). Only 6 % never applied surgical scoring systems on MM vertebral lesions. Clinical and radiological follow-up is routinely performed by 83 % of the participants, mostly by MRI (62 %), and less frequently by CT (12 %) or X-Ray (16 %), however only in 46 % at defined intervals (every 3–6 months: 43 %, >6 months: 3 %, when clinical status changes/at case-based intervals: 29 %, never: 17 %). Universally, 89 % would choose “less invasive” surgical strategies in MM than in similar lesions related to metastatic spine disease. However, 35 % of the participants would consider 360° reconstructive spine surgery using a combined anterior-posterior approach under certain circumstances (overview of participant's answers: Fig. 2A–H, Odd's Ratio of factors associated with the participant's answers: Fig. 3A-B). By calculating Odd's Ratio for factors influencing decision-making (Fig. 3A and B), the wide confidence intervals for some factors indicate a high variability in responses, while the routine discussion of spinal tumor cases with a spine surgeon and the routine application of scoring systems seem to shift opinions towards surgical management.

### 3.3 Treatment choices according to fictional case examples

**Fig. 4:**
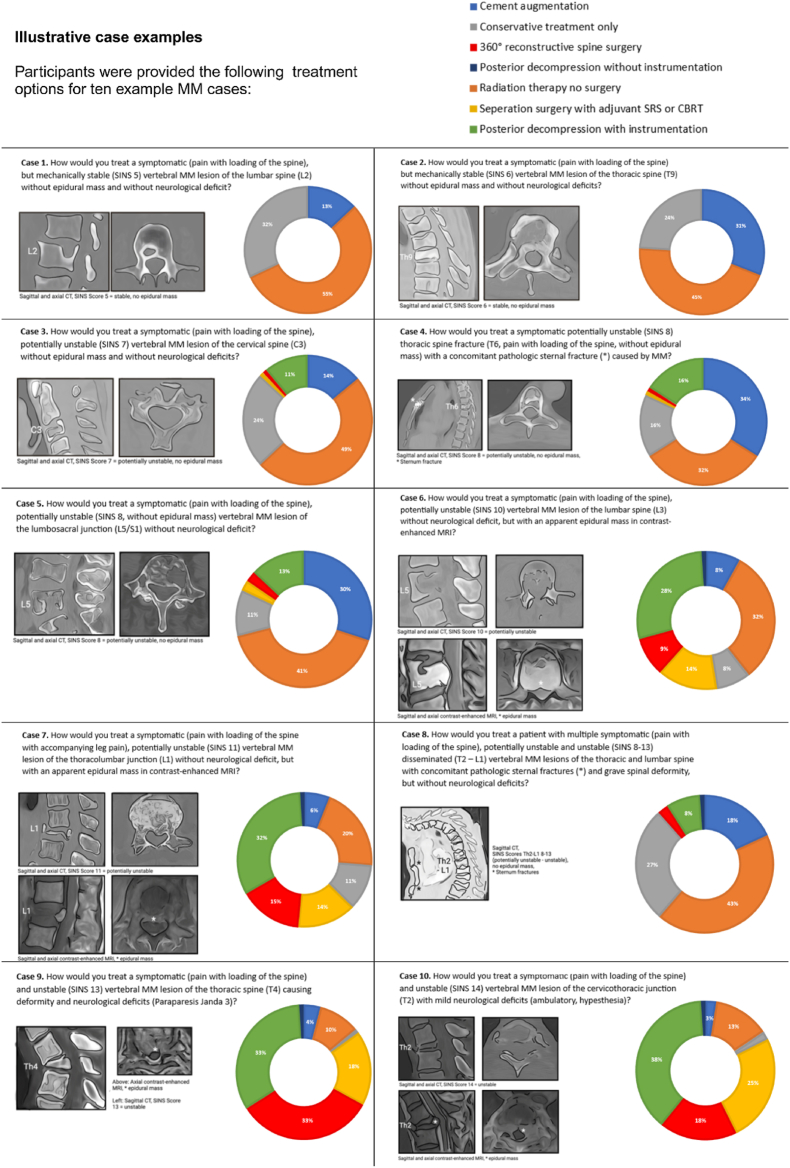
Ten exemplary, fictional case examples to assess the participant's perceived treatment standards for Multiple Myeloma vertebral column lesions, with the expert's treatment choices displayed in a tart chart. Values are given in %, with n = 51 equaling 100 %.

To assess the systematics of perceived treatment standards, ten fictional exemplary case examples of MM vertebral column lesions were created, with seven possible standardized treatment scenarios each to choose from. The ten exemplary case examples covered singular or disseminated lesions, with or without epidural infiltration, different regions of the spine (cervical, thoracic, lumbar/sacral), different symptoms (no symptoms, pain, neurological deficits), and different implications for spinal stability. Two case examples comprised of additional pathological sternal fractures (Table 1). In every case example, the SINS of the index lesion was given as additional information. Furthermore, every case example contained an axial and sagittal illustration of CT or MRI of the lesion. Fig. 4 displays the case examples, and the expert's respective treatment choices.

**Table 1:** Summary of clinical and radiological characteristics of the 10 case examples.

	Location	SINS	Clinical Symptoms	Other information
**Case 1**	Mobile spine (L2)	Stable	Vertebral pain	
**Case 2**	Semi-rigid spine (Th9)	Stable	Vertebral pain	
**Case 3**	Mobile spine (C3)	Pot. unstable	Vertebral pain	
**Case 4**	Semi-rigid spine (Th6)	Pot. unstable	Vertebral pain	Sternal fracture
**Case 5**	Junctional spine (L5)	Pot. unstable	Vertebral pain	
**Case 6**	Junctional spine (L5)	Pot. unstable	Vertebral pain	Epidural mass
**Case 7**	Junctional spine (L1)	Pot. unstable	Vertebral and leg pain	Epidural mass
**Case 8**	Disseminated (C-L/S)	(Pot.) unstable	Vertebral pain	Sternal fracture, spinal deformity
**Case 9**	Semi-rigid spine (Th4)	Unstable	Vertebral pain, paraparesis, not ambulatory	Deformity, epidural compression, neurological deficits
**Case 10**	Junctional spine (TH2)	Unstable	Vertebral pain, hypesthesia, ambulatory	Epidural compression, neurological deficits

## 4 Discussion

This is the first study investigating variations in international treatment approaches for MM vertebral column lesions, as reported by international, multidisciplinary experts in this field. The study‵s principal findings are that most experts would treat MM vertebral lesions less invasively than lesions from spinal metastatic disease or even recommend a non-surgical treatment strategy in general, although 74 % would consider neurological deficits as a strong indicator for surgery. The results of this survey provide guidance toward clinical decision-making related to MM vertebral lesions, enable future efforts at the development of care pathways and identify areas for future research.

Overall, the wide confidence intervals for certain factors indicate variability and uncertainty in responses. The discussion of tumor cases with a spine surgeon and the routine application of scoring systems are associated with a greater tendency toward surgical treatment. Conversely, when oncologists are primarily responsible for treatment decisions, they are more likely to classify MM vertebral lesions as a non-surgical condition compared to spine surgeons or radiation oncologists. Additionally, higher clinical experience and greater involvement of spine surgeons seem to reduce differentiation in the treatment of MM patients versus those with spinal metastases from solid tumors, indicating a more standardized approach in experienced surgical settings.

This variability in decision-making was also evident when experts were presented with fictive case examples. While neurological function played a key role in determining treatment invasiveness, lesion location, spinal stability, and epidural tumor infiltration were also influential factors. However, treatment strategies remained heterogeneous, with no clear correlation between preferred approaches and factors such as geographical location, clinical background, the specialty in charge, interdisciplinary case discussions, or the routine use of surgical scoring systems. This highlights the absence of standardized treatment framework for patients with MM vertebral lesions.

To further assess the impact of clinical and radiological characteristics on treatment decisions, fictive case examples were designed to vary in key aspects such as anatomical location, spinal stability, and accompanying symptoms like pain or neurological deficits. Spinal stability played a significant role, as cases with higher Spinal Instability Neoplastic Score (SINS) but without epidural mass or neurological deficits were more likely to be treated with cement augmentation or instrumentation, whereas SINS-stable lesions were often managed non-surgically. The presence of an epidural mass, however, consistently led to more invasive surgical approaches, such as decompression and instrumentation, rather than cement augmentation or conservative treatment. This effect was even more pronounced in cases with neurological deficits, which emerged as the strongest factor influencing surgical decision-making. Nonetheless, even in such cases, treatment choices varied widely, with some experts still favoring non-surgical management.

This heterogeneity in surgical decision-making highlights the lack of standardized treatment protocols for MM vertebral lesions. While studies on surgical management in MM are limited (Kyriakou et al., 2019; Osterhoff et al., 2023; Julka et al., 2014; Guzik, 2017; Roland et al., 2002; Khan et al., 2014; Tancioni et al., 2010; Rao et al., 2006), most evidence supports the use of vertebroplasty or kyphoplasty for pathological fractures to improve pain and clinical outcomes (Kyriakou et al., 2019; Audat et al., 2016; Schwarz et al., 2020), with low complication rates. In contrast, while timely surgical decompression is well-established for metastatic epidural compression (Cole and Patchell, 2008; Patchell et al., 2005; Barzilai et al., 2018), comparable evidence for MM-related vertebral disease remains scarce (Hubertus et al., 2025). The observed variability in surgical indications and approaches underscores the urgent need for standardized guidelines to optimize patient care and ensure consistent decision-making (Bilsky and Barzilai, 2021; Zijlstra et al., 2023b; Shi et al., 2024; Milavec et al., 2020b).

This goal is especially important as MM systemic therapy has distinctly evolved over the past two decades, now with promising immunotherapies available, including the use of CAR T-cell therapy or bispecific antibodies, as well as more extensive combinatorial therapies, thus further improving the patients' overall clinical outcome and prognosis (Rafae et al., 2024).

### 4.1 Limitations

With a total of 51 international experts from 14 countries and five continents represented in this study, there nevertheless exists a selection bias since most of these experts originate from North America and Europe. A response rate of 78 % to this survey was decent, however a higher rate would have been desirable. With study circulation to the target group of the AO Spine Knowledge Forum Tumor, an international expert group was addressed, which was not as broad or international as would have been a broader target group like AO Spine itself. However, the goal of this study was to firstly assess the opinions of clinically leading experts in the field, while further validation could be assessed in the future by circulation towards broader target groups.

Moreover, the provided case examples do not contain additional, clinically important information about patients‵ age and general health status, even though these factors were found to significantly influence surgical outcome in MM in previous studies and would likely influence treatment decisions (Shi et al., 2024). While this design choice simplifies the discussed case examples and increases generalizability, it also limits the variability of clinical decision-making. Furthermore, the experience of treating MM patients of each participant is not assessed, and a high variability is to be expected, even within the selected international expert group.

## 5 Conclusions and Outlook

The results of this international survey display the current international treatment approaches for MM vertebral column lesions and emphasize the lack of standardized treatment guidelines. While the participating experts generally agreed towards a more restrained and less invasive management of MM patients as compared to metastatic spine disease, the applicability of surgical scores, standards for clinical and radiological follow-up, and indications for surgery, as well as the selected surgical strategies for MM vertebral lesions varied widely. The provided fictional case examples revealed possible clinical and radiological factors that led the experts towards the choice of a more invasive treatment strategy, such as signs of spinal instability, the presence of an epidural mass, or the occurrence of neurological deficits. As standardized treatment guidelines for MM vertebral column lesions are desirable to improve therapeutic management and clinical outcome, future prospective studies investigating the influence of different treatment strategies on patient-related outcome measures, patients' long-term outcomes and the cost-effectiveness of different treatment strategies and are key to provide evidence towards achieving this goal.

## Contributions

JSO and VH design the study concept. All authors contributed to the study conception and design. Material preparation and data collection was performed by VH and EvB. Medical illustrations were created by LSF. JSO and PV supervised and monitored data collection. Data analysis was performed by VH, EvB and JSO. VH and EvB contributed to figure design. VH, JSO, EvB, PV, AMF, CE, HM and CB are part of the M2Spine Study Group. The manuscript was written by VH and JSO. PV and MGF critically revised the manuscript. All authors revised the manuscript and read and approved its final version.

## Competing interests and funding:

No funding was received for this study. The logistics of the survey dissemination were supported by AO Spine, and the AO Spine Knowledge Forum Tumor. VH is funded by the Berlin Institute of Health (BIH) Charité Clinician Scientist Program. JSO is a Clinical Fellow of the Stiftung Charité. MGF is the Robert Campeau Family Foundation/Dr. Charles Tator in Brain and Spinal Cord Research

## Declaration of competing interest

The authors declare that they have no known competing financial interests or personal relationships that could have appeared to influence the work reported in this paper.

## Abbreviations:

C – Cervical vertebra, CBRT – Conventional Beam Radiation Therapy, CT – Computed Tomography, IMWG - International Myeloma Working Group, KPS – Karnofky Performance Scale, L – Lumbar vertebra, MM – Multiple Myeloma, MRI – Magnetic Resonance Imaging, OR – Odd's Ratio, S – Sacral spine, SINS – Spinal Instability Neoplastic Score, SORG – Skeletal Oncology Research Group, SRS – Stereotactic Radiosurgery, Th = Thoracic vertebra.
